# Age-related hearing loss and balance disorders: analysis of interactions and clinical implications in older persons. Systematic review and meta-analysis

**DOI:** 10.3389/fragi.2025.1752488

**Published:** 2026-01-21

**Authors:** Andrea Frosolini, Leonardo Franz, Leonardo Calvanese, Valeria Caragli, Daniela Parrino, Cristoforo Fabbris, Giulio Badin, Michael Negrisolo, Roberta Cenedese, Lisa Doretto, Giuseppe Brescia, Cosimo de Filippis, Elisabetta Genovese, Elisabetta Zanoletti, Gino Marioni

**Affiliations:** 1 Maxillofacial Surgery Unit, Department of Medical Biotechnologies, University of Siena, Siena, Italy; 2 Phoniatrics and Audiology Unit, Department of Neuroscience DNS, University of Padova, Treviso, Italy; 3 ENT Unit, Surgical Department, Ospedali Riuniti Padova Sud, Monselice-Padova, Italy; 4 Department of Medical and Surgical Sciences for Children and Adults, Otorhinolaryngology Unit, University of Modena and Reggio Emilia, Modena, Italy; 5 Department of Otorhinolaryngology Head and Neck Surgery, ASST Sette Laghi, Ospedale di Circolo e Fondazione Macchi, Varese, Italy; 6 Otolaryngology Unit, Department of Neuroscience DNS, University of Padova, Padova, Italy

**Keywords:** age-related hearing loss, balance disorders, cochlear implant, falls, hearing aids, older persons

## Abstract

**Introduction:**

Age-related hearing loss (ARHL) frequently coexists with balance disorders in older persons, but the mechanisms and rehabilitative leverage of this association remain unsettled. We synthesized evidence on interactions between ARHL and vestibular/gait dysfunction, quantified mobility and fall outcomes, and appraised clinical implications for prevention.

**Methods:**

Following PRISMA and a registered PROSPERO protocol, we searched PubMed (MEDLINE), Scopus and Web of Science Core Collection. Inclusion required adults ≥65 years, ARHL and quantitative vestibular/balance outcomes. Forty studies met the criteria. Vestibular pooling was infeasible due to heterogeneous designs and metrics. In quantitative analysis, continuous outcomes were meta-analysed as standardized mean differences (SMD) (Hedges g) using random effects (REML) for ARHL–control contrasts and a fixed effect for within-participant Hearing Aid (HA) ON–OFF contrasts. For falls, we pooled Odds Ratio (OR) with REML.

**Results:**

Five out of seven studies linked ARHL to vestibular impairment. Across six studies, ARHL was associated with slower Timed Up to Go (SMD = −0.679), yet meta-regression showed systematic bias from age imbalance (−0.036 SMD per year older in HL), and the adjusted intercept was not significant. In three HA ON–OFF studies, static posturography improved with amplification (g = 0.459). The falls meta-analysis (k = 4; follow-up 12–60 months) showed higher fall odds with ARHL (OR = 1.55).

**Discussion:**

Age is a dominant driver of mobility, but ARHL contributes modifiable risk through sensory and cognitive-motor pathways. Consistent improvements with HA and converging gait data support integrating auditory rehabilitation—alongside vestibular/sensory-integration training—into multimodal fall-prevention strategies. Standardized protocols and age-balanced trials are priorities to refine effect estimates and clarify mechanisms.

## 1 Introduction

Sensory function integrity reflects the nervous system’s ability to receive, process, organize and integrate the information recognized by multiple sensory modalities, including the senses of sight, hearing, smell, taste, touch, movement, gravity and posture (Kutlu et al., 2024). Sensory skills allow humans to experience and react to their surroundings; therefore, an optimal and effective multisensory integration is essential for strengthening environmental adaptation (Kutlu et al., 2024; Brown et al., 2001). Among higher-order functions relying on multisensory integration, postural control and balance are particularly sensitive to sensory decline. Accordingly, growing evidence has suggested that sensory impairments, such as hearing loss (HL), can compromise balance and postural stability and increase the risk of falls (Lavie et al., 2024; Jiam et al., 2016). Moreover, untreated HL negatively impact many aspects of life – cognitive status, loneliness, depression and reduced quality of life (Lavie et al., 2024).

Both HL and balance disorders (BD) are common among adults aged over 60 years, with a reported prevalence around 20%–30% and 60%, respectively (Roth et al., 2011; Semenov et al., 2016). Nonetheless there is limited knowledge about the complex interplay between HL and BD (Zuniga et al., 2012; Riska et al., 2022; Teplitxky et al., 2023), thus further investigation into proper audiological assessments and interventions for the older persons are required. Several theories have been proposed to explain the relationship between HL and BD. Primarily, HL and dizziness are the results of a common age-related deterioration of both anatomical and physiological cochlear-vestibular functions. The human auditory and vestibular systems are located in the inner ear; therefore, any deterioration of these structures due to aging could contribute to postural instability (Viljanen et al., 2009a; Kurtaran et al., 2016). From an embryological point of view, the cochlea and saccule develop from the same origin in the membranous labyrinth, which is innervated by the inferior portion of the vestibular nerve. Furthermore, the saccule plays the role of an acoustic-sensitive organ in lower species; therefore, as a vestibular-sensitive organ, it can be considered as a late development in humans (Kurtaran et al., 2016). Chronic noise exposure, along with molecular mechanisms such as inflammation, ototoxicity, oxidative stress and genetic factors have been proposed as common factors that link cochlear and vestibular pathophysiology (Paplou et al., 2023). Moreover, HL can directly affect balance by reducing the auditory input. Postural stability relies on sensory inputs, including visual, vestibular and somatosensory systems, and auditory information has been demonstrated to potentially contribute to balance by acting as auditory biofeedback (Kurtaran et al., 2016; Vitkovic et al., 2016). The decline in listening quality increases the ‘listening effort’ required to process and comprehend auditory signals reallocating cognitive resources toward auditory processing and thereby reducing those available for postural control, gait regulation, and other balance-related tasks (Wang et al., 2024; Kim et al., 2025). Therefore, patients with significant HL use much of their cognitive capacity for hearing, leaving less available for balance control (Kim et al., 2025). Furthermore, individuals with asymmetric hearing have a higher postural instability risk compared to those with symmetric hearing (Wang et al., 2024). Sound localization depends on interaural differences in signal timing and intensity. Thus, individuals with asymmetric hearing may experience greater difficulties in localizing sound sources, consequently impairing their postural stability (Wang et al., 2024).

The objective of this review is to systematically analyse the existing literature on auditory and vestibular functional decline in the older persons, investigating their mutual interactions, associated health outcomes, and implications for clinical management and preventive strategies. We aim to provide evidence-based recommendations for integrated clinical practice and public health policies.

## 2 Materials and methods

### 2.1 Protocol registration and research questions

This systematic review protocol was developed following the Preferred Reporting Items for Systematic Reviews and Meta-Analyses (PRISMA) guidelines and registered on the PROSPERO database (registry number PROSPERO 2025 CRD420251019918). The research question was formulated as follows: “What are the interactions between auditory and vestibular functional decline in older adults, their associated health outcomes, and implications for clinical management and preventive strategies?” and structured according to Patient/Population/Problem, Intervention, Comparison, and Outcome (PICO) criteria (Vallamchetla et al., 2025).

### 2.2 Search strategy and eligibility criteria

A comprehensive search was conducted in three bibliographic databases—PubMed (MEDLINE), Scopus, and Web of Science Core Collection (WOS) —from database inception to 31 March 2025 (final search conducted on 31 March 2025). These sources were selected a priori to maximize coverage while ensuring reproducibility. In addition, reference lists of included studies and relevant reviews were screened to identify any potentially eligible articles not captured by database indexing. Used keywords included “hearing loss,” “vestibular dysfunction,” “balance disorders,” “aging,” “elderly,” “falls,” and combinations thereof.

Studies were eligible for inclusion if they met all the following criteria: i) Included adults aged ≥65 years (either in the whole population or in identifiable subgroups [this age represents the internationally accepted definition of “older adults” in geriatric medicine (e.g., WHO guidelines]) experiencing age-related HL (presbycusis) diagnosed through pure tone audiometry; ii) original clinical articles with quantitative data; iii) explicit investigation of the interaction or association between auditory functions, and their impact on vestibular function, balance performance or fall risk; iv) English as publication language. Interventions or exposures of interest included audiological rehabilitation strategies such as hearing aids (HA), cochlear implants (CIs) and auditory training; vestibular rehabilitation programmes; and combined auditory-vestibular or sensory integration therapies. Studies were also considered if they examined relevant exposures such as underlying audiological pathologies. When present, the controls included healthy older adults without hearing or balance disorders, no intervention or standard care, or alternative rehabilitative interventions.

Studies were excluded if they met any of the following criteria: i) Population aged <65 years or no subgroups’ data to be isolated from over-65 patients; ii) focused on acute conditions or neurological diseases unrelated to aging; iii) studies based on animal models or other preclinical investigations, reviews, editorials, commentaries, or case reports, conference abstracts without full data and other qualitative studies without quantitative measurements; iv) interventions not of interest: studies involving surgical, pharmacological, or rehabilitative interventions unrelated to auditory or vestibular function; investigations focused on acute trauma, inflammatory disease, or non-sensory medical conditions.

### 2.3 Study selection and eligibility criteria

Four independent reviewers (LC; VC; MN; AF) conducted the initial screening based on titles and abstracts, applying pre-defined inclusion and exclusion criteria. Articles selected underwent full-text screening to confirm eligibility. Studies without clear methodological rigour, lacking relevant outcomes, or with incomplete data were ruled out. Disagreements during the screening process were resolved by consensus or consultation with a senior author.

### 2.4 Data extraction and quality assessment

Data extraction was independently performed by the same 4 independent reviewers (LC; VC; MN; AF) using a standardized extraction form. Extracted data included: authors, publication year, study design, demographic details with focus on tendency and dispersion measures of age, methods of auditory and vestibular/gait assessment, key outcomes, findings and clinical implications. If manuscripts lacked sufficient data, they were excluded during this phase and the reasons have been reported within the Results section. The quality of studies was evaluated using appropriate Cochrane risk-of-bias tools based on study design (Sandoval-Lentisco et al., 2024). Discrepancies were settled through consensus.

### 2.5 Data synthesis and analysis

Extracted data were synthesized using a structured narrative synthesis: studies were grouped a priori by outcome domain (vestibular function, balance/gait performance, fall outcomes, and rehabilitative interventions), and findings were summarized in terms of direction, consistency, and clinical relevance. Where appropriate, a quantitative summary was performed. For continuous outcomes, time up and go (TUG), we used standardized mean differences (SMD) as the summary effect. Between-group comparisons (HL vs. controls) were pooled with a random-effects model using the Restricted Maximum Likelihood (REML) estimator to account for between-study variability; effects were coded so that negative values indicated worse performance in the HL group. For falls (binary outcomes), when studies reported falls by hearing status, we used reported odds ratios (ORs) and 95% confidence intervals where available; otherwise, we reconstructed ORs and standard errors from 2 × 2 tables. To preserve independence, we retained one contrast per study a priori. We prioritized “any fall” over alternative endpoints and 12-month recall. Effects were coded so that OR>1 indicates higher odds of falling in the age-related hearing loss (ARHL) group. As robustness checks, we repeated the model after excluding the non-12-month window and the ≥2-falls endpoint. For within-participant ON–OFF hearing-aid contrasts, the primary model was fixed-effect (inverse-variance weighting) because k was small (k = 3) and τ^2^ was poorly estimable. Effects were coded so that positive values indicated better performance with HA ON (ON–OFF). For ON–OFF studies reporting multiple stances/metrics, we pre-specified a single challenging static stance (eyes-closed on foam or closest analogue) and one primary posturography metric per study to minimize multiplicity. A two-sided p < 0.05 was considered statistically significant. Heterogeneity was assessed with the Q-test, I^2^, and τ^2^. Outlier/influence diagnostics used studentized residuals and Cook’s distance. To explore the influence of age imbalance between groups in the HL-vs-control synthesis, we performed a mixed-effects meta-regression using the mean age difference (HL–control) as a continuous moderator. For the ON–OFF analysis (k = 3), meta-regression was considered exploratory only. Potential small-study effects were examined visually (funnel plots) and statistically (Egger’s regression, Begg–Mazumdar rank correlation, Rosenthal’s fail-safe N). Analyses were conducted in jamovi (version 2.3; The jamovi project, Sydney, Australia; accessed 29 July 2025).

## 3 Results

### 3.1 Search results, general characteristics and quality assessment of included studies

**FIGURE 1:**
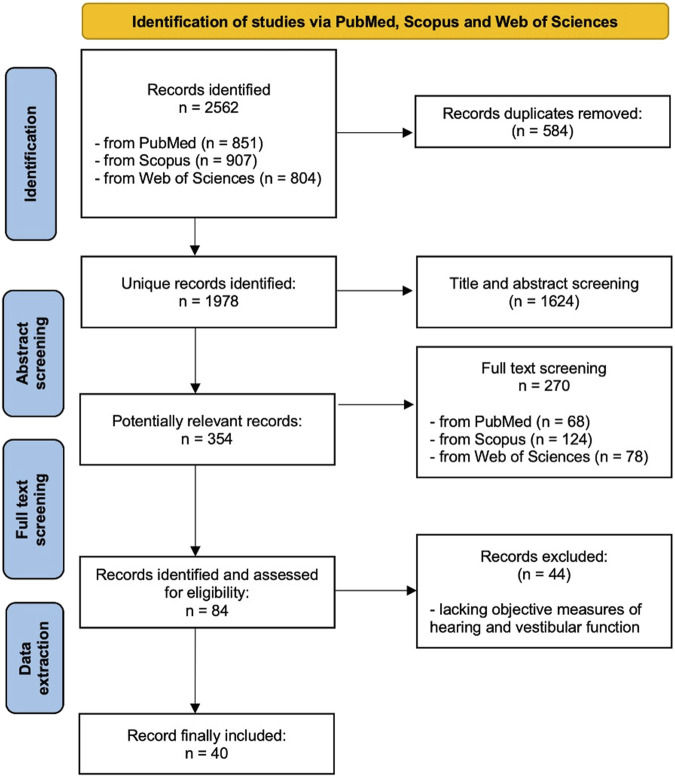
Prisma flow diagram.

The initial database search yielded a total of 2,562 records across three major sources: Scopus (n = 907), WOS (n = 804), and PubMed (n = 851). After removing duplicates, a total of 1,978 unique records were identified. These entries underwent title and abstract screening to assess relevance according to the predefined inclusion and exclusion criteria outlined in the PROSPERO protocol, thus identifying 354 potentially relevant studies. After full-text screening, 270 articles (124 from Scopus, 78 from WOS, and 68 from PubMed) were excluded due to the following reasons. Irrelevant outcomes [e.g., did not assess hearing, vestibular, or gait parameters): 71 articles (26.3%)]; wrong population [e.g., non-elderly subjects, paediatric cohorts, or undefined age groups: 48 articles (17.8%)]; inadequate study design [e.g., narrative reviews, editorials, case reports, or conference abstracts: 44 articles (16.3%)]; missing or unclear methodology [e.g., small sample size, no control group, or lacking statistical analysis: 29 articles (10.7%)]; non-English language or full text not accessible [22 (8.1%)]; cognitive or psychosocial focus without audiological/vestibular relevance [18 (6.7%)]; combined populations without age-stratified data [16 (5.9%)]; other reasons (e.g., focused solely on device engineering, occupational noise, or unrelated imaging findings: 22 (8.1%)]. The final pool of articles that underwent data extraction consisted of 84 manuscripts. After data extraction additional 44 manuscripts were excluded as lacking objective measures on hearing or vestibular function. Figure 1 presents the PRISMA flow diagram, while Tables 1-3 summarize the general characteristics of the 40 included studies based on the PICO criteria and their assessed risk of bias.

**TABLE 1:** Studies addressing ARHL and vestibular function.

First author, year	Country	Design	Risk of Bias	Population (n; definition; mean age)	Comparison	Measure	Outcome
Azevedo et al. (2022)	Portugal	RCS	M/S	40; ENT patients; 70	Young adults	TCR	No significant TCR differences between groups; significant correlation between PTA and TCR
Gabriel et al. (2022)	Canada	CS	L	37; healthy elderly; 70.4	Young adults	vHIT; cVEMP; vestibular threshold	Older adults had higher heave and pitch detection thresholds
Kurtaran et al. (2016)	Turkey	CS	L	63; HL patients; 67.2	Age-matched non-HL	cVEMP	Significant differences between groups in VEMP latency and amplitude
Nayak et al. (2022)	United States	RCS	M	149; CI candidates; 74.4	NA	TCR	High prevalence of abnormal preoperative vestibular testing
Onn et al. (2021)	Malaysia	CS	M	135; ENT patients; 65.3	NA	vHIT	Prevalence of vestibular dysfunction is independently associated with ageing and presbycusis
Teplitxky et al. (2023)	France	CS	L	53; ARHL; 84.2	Subgroup: Expected ARHL *vs* untimely ARHL	VNG; vHIT	No significant associations between untimely ARHL and presby-vestibulopathy, or between the PTA high and presby-vestibulopathy
Zuniga et al. (2012)	United States	CS	M	51; Elderly volunteer; 77.1 (estimate)	NA	htDVA; cVEMP, oVEMP.	PTA correlated with saccular (not utricular) function

Abbreviations: CI: cochlear implant; CPA: Cerebello-pontine angle; CS: Cross-sectional study; cVEMP: cervical vestibular evoked myogenic potential; ENT: Ear Nose and Throat; HL: Hearing Loss; KNHNS: Korean National Health and Nutrition Survey; L: low; M: moderate; MD: Meniere disease; oVEMP: cervical vestibular evoked myogenic potential; PTA: pure tone average; RCS: Retrospective cohort study; S: serious; SNHL: Sensory neural hearing loss; TCR: Total Caloric Response; VEMP: vestibular evoked myogenic potential; htDVA LogMAR: dynamic visual acuity during head thrusts logarithm of the minimum angle of resolution; vHIT: video Head Impulse Test; VNG: Videonystagmography.

**TABLE 2:** Studies addressing ARHL and gait.

First author, year	Country	Design	Risk of Bias	Population (n; definition; mean age)	Comparison	Measures	Outcome
Basta et al. (2023)	Germany	CS	M	16; Elderly; 67.1	NA	Sway during walking	Auditory information significantly improved postural control during walking
Bruce et al. (2019b)	Canada	CS	M	32; ARHL; 70.7	Age-matched non-HL; young adults	DGI; Sit to stand Task; ABC scale; custom made perturbation plat- form	ARHL group demonstrated greater dual-task costs in noise leading to worst balance performer
Cosetti et al. (2025)	United States	CS	M	20; Elderly HL; 69)	Age-matched non-HL	TUG; DHI; ABC scale	Those with BHL performed slower on sub-component analysis of the instrumented TUG compared with age-matched control
Chongvisal et al. (2019)	Thailand	CS	M	828; Elderly; 69.6	Subgroup analysis	Survey	All age groups reported dizziness and vertigo but the elderly over 75 years reported more falls with a statistical significance
Ciquinato et al. (2020)	Brazil	CS	M	247; ELLO Project: Study on Aging and Longevity; 68.4	Subgroup: SNHL *vs* NH	Posturography	Elderly individuals with HL presented greater instabilities in the posturographic evaluation
Gorecka et al. (2021)	Norway	CS	M	42; MCI geriatric patients; 71.1	Age-matched controls	OptoGait System	Demands on attentional control dependent on hearing acuity affects gait negatively in healthy older adults and aMCI individuals
Kamil et al. (2016)	United States	PCS	M	1168; community-dwelling elderly HL; 74.3 (estimate)	Age-matched non-HL	Falls	HL is independently associated with greater odds of falling over time in older adults.
Koh et al. (2015)	Republic of Korea	CS	M	46; elderly; 76	Subgroup: SNHL *vs* NH	TUG; OLST	Significant correlation between hearing loss and static balance
Kolasa et al. (2023)	Norway	CS	L	4101; HUNT4;	NA	SPPB	Increased hearing threshold is associated with poor physical performance
Kolasa et al. (2024)	Norway	CS	M	50; ARHL and HA; 76.2	Age-matched non-HL	SPPB; Posturography; DHI	HL was associated with worse physical performance as measured by SPPB, postural sway, and gait variability
Lau et al. (2016)	Canada	CS	M	8; bilateral HL and HA; 73.3	Age-matched non-HL	Kinematic parameters (TUG)	HL group demonstrated more overall stride time variability than the NH group
Mamo et al. (2023)	United States	PCS	M	144; PACE; 74	NA	Falls	More falls over 3 years among participants with HL at baseline
Mold et al. (2024)	United States	PCS	M	637; OKLAHOMA Studies cohort; 73.9 (estimate)	Subgroup: no HL; mild; moderate; severe	Tinetti Balance Scale; 50 feet time walk	ARHL is associated with reduced life expectancy, probably mediated through an adverse impact on balance
Morris et al. (2023)	United States	CS	M	14; elderly BHL;	UHL; NH	TUG; ABC; Visual Vertigo Analog Scale; Four-Square Step Test	Adults with BHL demonstrated a pattern of balance impairment
Purchase-Helzner et al. (2004)	Germany	PCS	L	6480	NA	Falls	The age-adjusted annual fall rate did not differ significantly by hearing category, nor did the risk of incident fracture
Sakurai et al. (2021)	Japan	CS	M	107; Elderly volunteers; 76.5	Subgroup: HL; no HL	Gait speed stride length and stride time variability in walking; Falls	The association between HL and increased gait variability could jeopardize gait control during daily activities leading to increased risk of accidental falls
Sakurai et al. (2022)	Japan	RCS	M/S	810; Elderly (Otassha and REPRINTS studies); 74 (estimate)	Subgroup: normal; mild and moderate HL	Gait speed (m/s) in timed 5-meter walk; Falls	Interactions between gait performance and moderate hearing loss on both global cognition and the occurrence of falls
Sakurai et al. (2025a)	Japan	PCS	M	786; Elderly (Otassha study); 72.9	Subgroup: based on ARHL and Slow Gait	Gait speed (m/s) in timed 5 and 10 meters walk; Falls	Simultaneous presence of ARHL and SG significantly increased the risk of multiple falls, fall-induced fractures, and minor injuries
Szeto et al. (2021)	United States	CS	L	80; ENT outpatients; 73.7	NA	Gait parameters in 100m walking; DHI	Importance of auditory feedback for balance and coordination; right ear advantage for the influence of auditory feedback on gait
Viljanen et al. (2009b)	Finland	CS	L	423; FITSA; 68.6	Between female twins	Posturography; Falls	People with poor hearing acuity have a higher risk of falls, which is partially explained by their poorer postural control
Viljanen et al. (2009a)	Finland	PCS	L	434; FITSA; 68.6 (Estimate)	Subgroup: BEHL 0.5–4 kHz <21dB; BEHL 0.5–4 kHz >21dB	Walking speed, endurance and difficulty	Hearing acuity correlated with mobility, which may be explained by the association between impaired hearing and poor balance
Wollesen et al. (2018)	Germany; Australia	CS	M	73; Elderly volunteers; 71.2 (estimate)	Subgroup: no HL; Mild HL; Moderate/severe HL	Gait parameters in 10m walk	Walking speed was reduced accompanied by decreased step length and increased cadence in people with more severe hearing loss

ABC: Activities-Specific Balance confidence scale; BEHL: Better ear hearing threshold level; BHL: bilateral hearing loss CS: Cross-sectional study; DGI: Dynamic Gait Index; cVEMP: cervical vestibular evoked myogenic potential; FITSA: Finnish Twin Study on Aging; HA: Hearing aids; HL: Hearing Loss; HUNT4: North- Trøndelag Health Survey; L: low; M: moderate; MCI: Mild Cognitive Impairment; MD: Meniere disease; NG: Normal gait; NH: normal hearing; NHI: National Health Insurance; OLST: one-leg stance test; oVEMP: cervical vestibular evoked myogenic potential; PACE: Programs of All-Inclusive Care for the Elderly; PCS: Prospective cohort study; PTA: pure tone average; RCS: Retrospective cohort study; S: serious; SG: Slow gate; SPPB: Short Physical Performance Battery; SNHL: Sensory neural hearing loss; UHL: unilateral hearing loss; VEMP: vestibular evoked myogenic potential; htDVA LogMAR: dynamic visual acuity during head thrusts logarithm of the minimum angle of resolution.

**TABLE 3:** Studies addressing rehabilitative intervention.

First Author, Year	Country	Study Design	Risk Of Bias	Population (n; definition; age in years)	Intervention: administration	Measure	Outcome
Behtani et al. (2024)	Canada	CS	L/M	30; ARHL and HA; 69	HA: ON *vs* OFF	Posturography; modified clinical sensory integration in balance test (mCTSIB)	Beneficial impact on postural control when visual and somatosensory inputs were reduced.
Bruce et al. (2019a)	Canada	CT	M	42, Elderly; 68.05	Cognitive and Aerobic Training: simultaneous *vs* sequential paradigm	Sit-to-Stand task; Posturography	All participants improved on a measure of chair rises; there was no benefit to standing balance; advantage to Sequential training
Mohanathas et al. (2024)	Canada	CS	M	22; ARHL and HA; 75.1	HA: ON *vs* OFF	Posturography	Did not remarkably improve balance
Rumalla et al. (2015)	United States	CS	M	14; Elderly HL and HA; 77	HA: ON *vs* OFF	Romberg; Tandem	Significant improvement in balance and decreased risk of falling
Campos et al. (2023)	United States	CS	M	299; Elderly HL and HA; 73.8	HA: aided *vs* unaided; Inconsistent vs consistent users	Fall Risk Questionnaire (FRQ)	Lower odds of falling and reduction in fall risk
Mahmoudi et al. (2019)	United States	RCS	M	14109; Elderly HL and HA; 75.8	HA: aided *vs* unaided	Falls and fall-related injuries	Delay or prevention of fall-related injuries
Picciotti et al. (2024)	Italy	CT	M	15; ARHL and dizziness; 75.1	HA: baseline *vs* 1year fitting	Conley Scale; DHI	Reduced the risk of falls and improved the spatial memory
Sakurai et al. (2025b)	Japan	CT	M	10; ARHL; 81	HA: baseline *vs* 1year fitting	Gait (speed, time, length)	Improved gait performance
Soylemez et al. (2024)	Turkey	CS	M	30; Outpatients HL and HA; 73	HA: aided *vs* unaided *vs* younger healthy adults	TUG	Improved TUG in Dual task condition
Negahban et al. (2017)	IRAN	CS	L	47; Outpatient Audiology; 67.4	HA: ON *vs* OFF *vs* unaided	Posturography	Significant improvement in postural stability
Prasansuk et al. (2004)	Thailand	CT	L	215; Elderly reporting dizziness; 67.4	Cawthorne-Cooksey exercises: 20 weeks *vs* no treatment	Posturography	Helpful and acceptable: 90% satisfaction

Abbreviations: BHL: bilateral hearing loss CS: Cross-sectional study; CT: Clinical Trial; DGI: Dynamic Gait Index; cVEMP: cervical vestibular evoked myogenic potential; HA: Hearing aids; L: low; M: moderate; MD: Meniere disease; NG: Normal gait; NH: normal hearing; oVEMP: cervical vestibular evoked myogenic potential; PCS: Prospective cohort study; PTA: pure tone average; RCS: Retrospective cohort study; S: serious; SG: Slow gate; SNHL: Sensory neural hearing loss; UHL: unilateral hearing loss; VEMP: vestibular evoked myogenic potential; htDVA LogMAR: dynamic visual acuity during head thrusts logarithm of the minimum angle of resolution.

### 3.2 Qualitative synthesis

Hearing function was assessed using pure tone audiometry across all studies, with some research groups complementing it with speech audiometry (Teplitxky et al., 2023), tympanometry (Picciotti et al., 2024), or self-perceived measures such as the Hearing Handicap Inventory for the Elderly (Mohanathas et al., 2024) and the Speech, Spatial and Qualities of Hearing Scale (G et al., 2022; Morris et al., 2023). ARHL was operationalized in two ways: i) Age-normative thresholds benchmarked against age- and sex-specific percentiles, classifying hearing as “worse than expected for age” when ear-specific thresholds exceed the >90th, an approach used in 1/40 studies (Teplitxky et al., 2023); ii) absolute definition according to HL grading, an approach used in 39/40 studies (Zuniga et al., 2012; Viljanen et al., 2009a; Kurtaran et al., 2016; Picciotti et al., 2024; Mohanathas et al., 2024; G et al., 2022; Morris et al., 2023; Azevedo et al., 2022; Nayak et al., 2022; Onn et al., 2021; Basta et al., 2023; Bruce et al., 2019a; Bruce et al., 2019b; Cosetti et al., 2025; Chongvisal et al., 2019; Ciquinato et al., 2020; Gorecka et al., 2021; Kamil et al., 2016; Koh et al., 2015; Kolasa et al., 2023; Kolasa et al., 2024; Lau et al., 2016; Mamo et al., 2023; Mold et al., 2024; Purchase-Helzn et al., 2004; Sakurai et al., 2021; Sakurai et al., 2022; Sakurai et al., 2025a; Sakurai et al., 2025b; Szeto et al., 2021; Viljanen et al., 2009b; Wollesen et al., 2018; Behtani et al., 2024; Rumalla et al., 2015; Campos et al., 2023; Mahmoudi et al., 2019; Soylemez et al., 2024; Negahban et al., 2017; Prasansuk et al., 2004).

Seven studies, as shown in Table 1, explored vestibular dysfunction in individuals with ARHL (Zuniga et al., 2012; Teplitxky et al., 2023; Kurtaran et al., 2016; G et al., 2022; Azevedo et al., 2022; Nayak et al., 2022; Onn et al., 2021; Mold et al., 2024). The majority of these studies - five out of seven - concluded that ARHL was associated with poorer vestibular outcomes (Zuniga et al., 2012; Kurtaran et al., 2016; G et al., 2022; Azevedo et al., 2022; Nayak et al., 2022): two studies reported significant correlations between pure tone average (PTA) and caloric tests (Azevedo et al., 2022) or VEMPS (Viljanen et al., 2009a). Three research groups found significant differences in terms of VEMPS (Kurtaran et al., 2016; G et al., 2022) and vHIT (Onn et al., 2021) when comparing ARHL with age matched subjects with normal hearing. Instead, two studies did not find significant associations between ARHL and caloric responses or vHIT (Teplitxky et al., 2023; Onn et al., 2021).

A total of 22 articles examined the impact of ARHL on static and dynamic gait, as resembled in Table 2. Functional mobility was primarily evaluated through standardized assessments such as the TUG test (Mohanathas et al., 2024; Morris et al., 2023; Cosetti et al., 2025; Koh et al., 2015; Lau et al., 2016; Soylemez et al., 2024) and gait analysis (Basta et al., 2023; Bruce et al., 2019a; Gorecka et al., 2021; Kamil et al., 2016; Kolasa et al., 2023; Lau et al., 2016; Mold et al., 2024; Sakurai et al., 2021; Sakurai et al., 2022; Sakurai et al., 2025a; Szeto et al., 2021; Viljanen et al., 2009b; Wollesen et al., 2018). These evaluations consistently demonstrated that individuals with HL showed slower walking speeds, prolonged transition times and impaired dynamic balance compared to age-matched controls. Complementing these mobility-focused assessments, eight investigations also incorporated dual-task paradigms which explored the additional cognitive load imposed by concurrent auditory processing tasks during ambulation (Morris et al., 2023; Basta et al., 2023; Bruce et al., 2019a; Cosetti et al., 2025; Gorecka et al., 2021; Kolasa et al., 2024; Lau et al., 2016; Wollesen et al., 2018). These studies revealed that ARHL exacerbated cognitive-motor interference, leading to diminished postural control under complex task conditions. To further characterize postural stability, static and dynamic posturography is frequently employed (Marioni et al., 2013). These analyses frequently included measures such as centre of pressure (CoP) displacement and sway area, which consistently revealed greater instability in individuals with ARHL, especially when visual or somatosensory cues were limited (Bruce et al., 2019a; Ciquinato et al., 2020; Kolasa et al., 2024; Viljanen et al., 2009b). In addition to instrumented tests, clinical balance scales were applied to quantify equilibrium and fall risk in everyday contexts such as the Activities-Specific Balance confidence scale (Morris et al., 2023; Bruce et al., 2019a; Cosetti et al., 2025) and the Dynamic Gait Index (Bruce et al., 2019a). Lastly, fall events were captured using the Falls Efficacy Scale International (FES-I) and self-reported fall frequency (Viljanen et al., 2009a; Chongvisal et al., 2019; Kamil et al., 2016; Mamo et al., 2023; Purchase-Helzn et al., 2004; Sakurai et al., 2021; Sakurai et al., 2022; Sakurai et al., 2025a). Excluding Purchase-Helzner (Purchase-Helzn et al., 2004) who reported that the age-adjusted annual fall rate did not differ significantly by hearing category, all the other research groups (Viljanen et al., 2009a; Chongvisal et al., 2019; Kamil et al., 2016; Mamo et al., 2023; Sakurai et al., 2021; Sakurai et al., 2022; Sakurai et al., 2025a) found a correlation between ARHL and risk of falls.

The most frequently investigated intervention for ARHL was HA, as reported by nine studies (Picciotti et al., 2024; Mohanathas et al., 2024; Sakurai et al., 2025b; Behtani et al., 2024; Rumalla et al., 2015; Campos et al., 2023; Mahmoudi et al., 2019; Soylemez et al., 2024; Negahban et al., 2017) (see also Table 3). The majority of the available studies (Picciotti et al., 2024; Sakurai et al., 2025b; Behtani et al., 2024; Rumalla et al., 2015; Campos et al., 2023; Soylemez et al., 2024; Negahban et al., 2017) reported positive effects of HA on postural stability, gait performance, or fall risk reduction, using a variety of outcome measures including instrumented posturography, the TUG, and standardized fall-risk scales. Notably, Rumalla (Rumalla et al., 2015), Negahban (Negahban et al., 2017) and Behtani (Behtani et al., 2024) demonstrated an improved postural control in HA-ON vs. HA-OFF experimental conditions. In contrast, Mohanathas (Mohanathas et al., 2024) failed to observe significant balance improvements in the same experimental setting. Campos (Campos et al., 2023) and Mahmoudi (Mahmoudi et al., 2019) - the latter with over 14,000 participants - reported lower fall risk and delayed fall-related injuries among HA users. Picciotti (Picciotti et al., 2024) and Sakurai (Sakurai et al., 2025b) further indicated longitudinal improvements following 1 year of HA use.

Two studies investigated non–HA interventions aimed at improving balance in older adults with hearing impairment (Bruce et al., 2019a; Prasansuk et al., 2004). Prasansuk (Prasansuk et al., 2004) conducted a clinical trial involving 215 older adults who underwent a 20-week Cawthorne-Cooksey vestibular rehabilitation programme, first reported in the 1940s (Marioni, 2022). The intervention demonstrated high acceptability and was associated with notable improvements in posturographic measures of balance, supporting its utility in managing age-related balance deficits. In a clinical trial of 42 participants, Bruce (Bruce et al., 2019a) evaluated the effects of cognitive and aerobic training, delivered either simultaneously or sequentially. While both intervention paradigms led to improvements in chair rise performance, no significant benefit on standing balance was observed, and the sequential training protocol was slightly more advantageous.

### 3.3 Quantitative analysis

**FIGURE 2:**
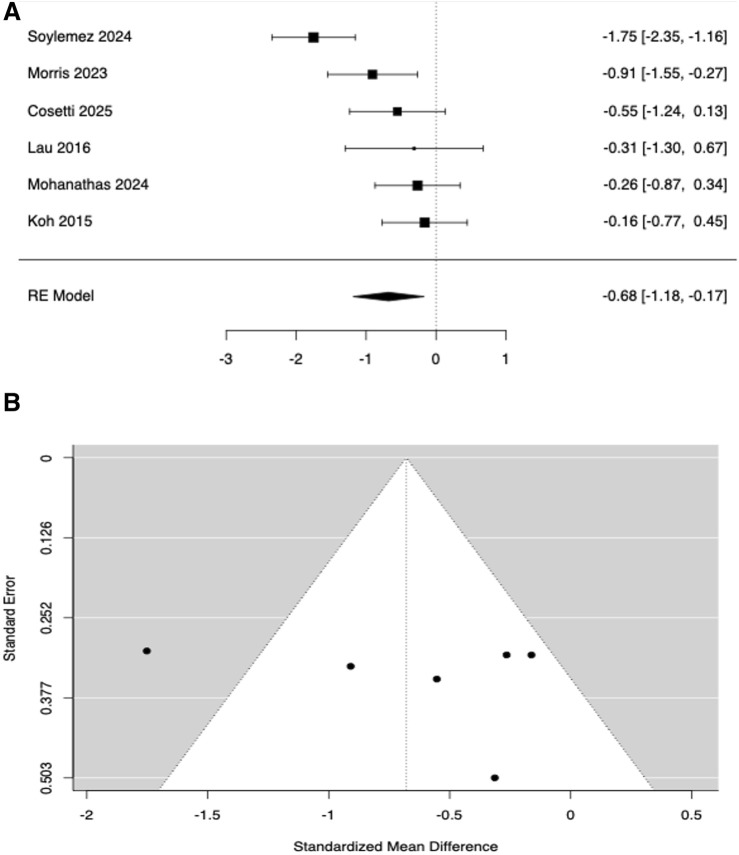
Forest **(A)** and funnel **(B)** plots for Timed Up to Go comparing age-related hearing loss (ARHL) vs. normal-hearing controls. Effects are Hedges g (negative = worse/slower in ARHL) pooled with a random-effects model.

**FIGURE 3:**
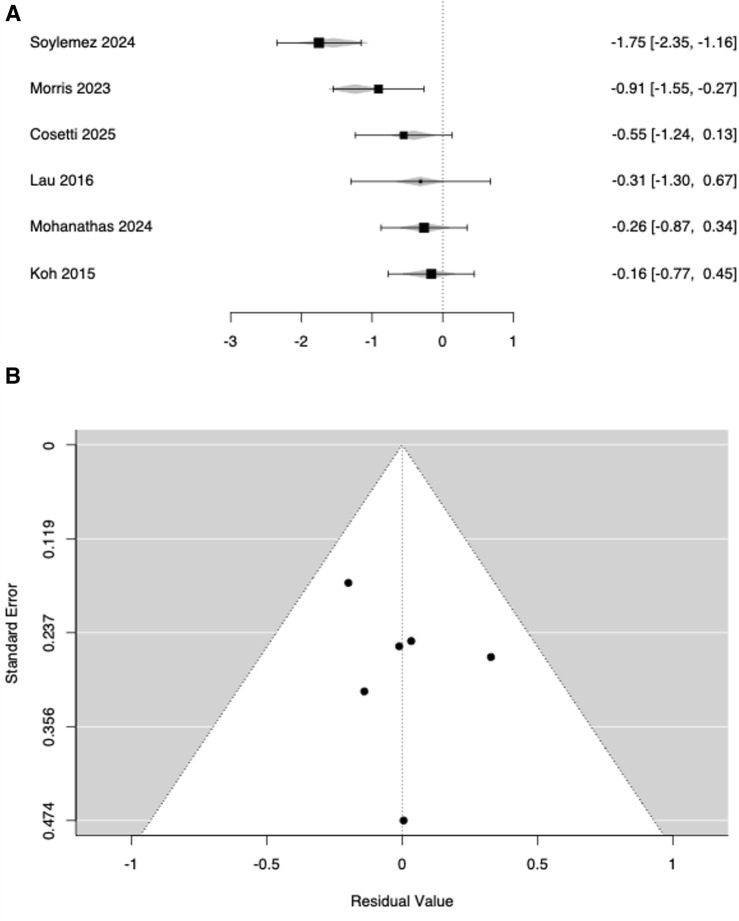
Meta-regression of Timed Up and Go effect sizes comparing ARHL vs. normal-hearing controls as a function of between-group age difference. Forest **(A)** and funnel **(B)** plots.

Six studies (Mohanathas et al., 2024; Morris et al., 2023; Cosetti et al., 2025; Koh et al., 2015; Lau et al., 2016; Soylemez et al., 2024) compared TUG performance in subjects with HL vs. controls without HL. Study-level SMDs ranged from −1.75 to −0.16, with all estimates favouring the control group (i.e., worse performance in HL). The random-effects model yielded a statistically significant average effect size of SMD = −0.679, indicating that individuals with HL exhibited worse dynamic balance than controls (Figure 2). Substantial heterogeneity was observed (Q (5) = 18.15, p = 0.003; I^2^ = 70.8%; Tau^2^ = 0.2771). The 95% prediction interval was −1.83 to 0.47, indicating that, although the mean effect was negative, true effects might vary and could be near zero or positive in some populations. Studentized residuals suggested one potential outlier (Soylemez et al., 2024) exceeding the Bonferroni-adjusted threshold (±2.64), whereas Cook’s distance showed no undue influence. To examine confounding by age, a mixed-effects meta-regression including the mean age difference (HL–control) as a moderator showed a significant association (estimate = −0.0363; Z = −4.07; p < 0.001; 95% confidence interval −0.054 to −0.019; Figure 3). Thus, for each 1-year increase in age imbalance (HL older than controls), the effect became ∼0.036 SMD units more negative, indicating systematic bias due to age differences. Clinically, this corresponded to ∼0.9 s slower TUG per 10-year age imbalance (assuming a representative pooled TUG SD ˜ 2.5 s). After adjusting for age difference, the intercept was not significant (SMD = −0.196, p = 0.294) and residual heterogeneity was eliminated (Tau^2^ = 0; I^2^ = 0%; Q (5) = 1.61, p = 0.808; R^2^ = 100%). No significant evidence of publication bias was detected (Begg–Mazumdar p = 0.71; Egger p = 0.937; Rosenthal’s fail-safe N = 49, p < 0.001). Trim-and-fill did not impute missing studies.

**FIGURE 4:**
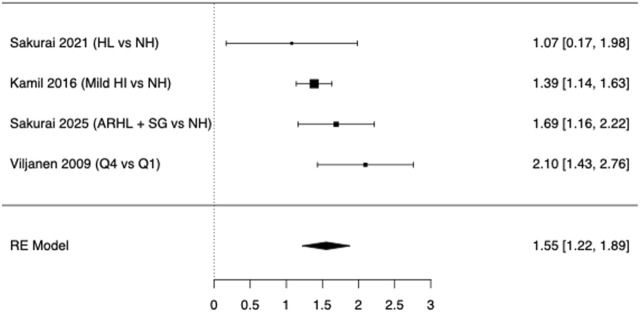
Forest Plot for fall at 12–60 months comparing ARHL with normal hearing (or, where quartiles were reported, the poorest hearing category vs. the best one).

Regarding fall risk in HL patients, we pooled k = 4 independent contrasts: three studies reporting 12-month “any fall” (Kamil et al., 2016; Sakurai et al., 2021; Viljanen et al., 2009b); one using a 5-year window (Sakurai et al., 2025a). Older adults with ARHL had 55% higher odds of falling than their normal-hearing counterparts, as shown in Figure 4 (OR = 1.55, 95% confidence interval 1.22–1.89, Z = 9.16, p < 0.001). Between-study heterogeneity was moderate (τ^2^ = 0.0458 [SE 0.0963], τ = 0.214; I^2^ = 39.6%; Q (3) = 5.25, p = 0.154). Small-study effects were not evident (Egger’s p = 0.897; Kendall’s τ p = 0.750), and the Rosenthal fail-safe N was 242, suggesting the result is unlikely to be explained by unpublished null findings.

**FIGURE 5:**
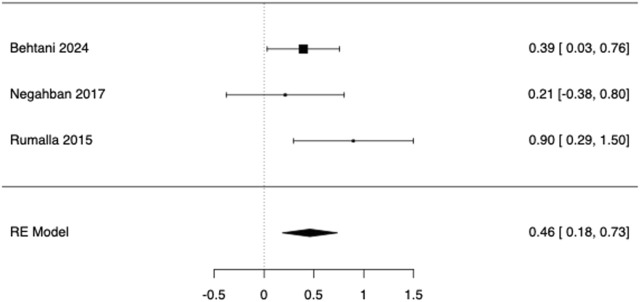
Forest plot of posturography outcomes in ARHL comparing Hearing Aid (HA) ON vs. OFF (within-participant). Effects are Hedges g (positive = better with HA ON) pooled with a random-effects model; stance pre-specified as eyes-closed on foam (or closest analogue).

Three within-participant contrasts comparing static balance with HA ON vs. OFF were synthesized (Behtani et al., 2024; Rumalla et al., 2015; Negahban et al., 2017). Study-level Hedges’ g ranged from 0.21 to 0.90, with all estimates favouring the ON condition (Rumalla (Rumalla et al., 2015) g = 0.895, SE = 0.307; Behtani (Behtani et al., 2024): g = 0.394, SE = 0.185; Negahban (Negahban et al., 2017): g = 0.212, SE = 0.302). The REML indicated a moderate improvement with hearing aids ON (pooled Hedges g = 0.459, SE = 0.140; 95% confidence interval 0.184–0.734; Z = 3.27; p = 0.001; k = 3; Figure 5). Between-study heterogeneity was not detected (Q (2) = 2.81, p = 0.245; I^2^ = 0%; τ^2^ = 0.00 [SE = 0.066]), so the REML summary coincided with the fixed-effect estimate. Given k = 3, small-study bias tests were treated cautiously and were not informative (Egger p = 0.743; Kendall’s tau p = 1.000; Rosenthal fail-safe N = 10). A two one-sided tests (TOST) procedure with pre-specified bounds of ±0.50 SMD was non-significant (Z = −0.290, p = 0.386), indicating that equivalence to ≤|0.50| SMD could not be concluded; the null-hypothesis test was significant (Z = 3.274, p = 0.001). With so few studies, prediction intervals and funnel/asymmetry diagnostics were not informative; meta-regression on age did not converge and was not interpreted.

## 4 Discussion

The WHO defines active aging as ‘the process of optimizing opportunities for health, participation, and security to enhance the quality of life as people age, allowing individuals to realize their potential for physical, social, and mental wellbeing throughout the life course’ (de Lima et al., 2024). Reduced mobility may lead to limitations in spatial awareness, depression and social isolation. ARHL, beside accelerating cognitive decline, appears to affect balance and ultimately fall risk, thus reducing the chances of retaining an active aging (Cosetti et al., 2025; Ivanenko and Gurfinkel, 2018; Rafoul et al., 2025; Wunderlich et al., 2024; Berge et al., 2019; Agmon et al., 2017; Foster et al., 2022; Ferrucci et al., 2012).

The available evidence that patho-physiologically and clinically correlates of such associations is discussed in detail below.

### 4.1 ARHL effects on vestibular system

A meta-analysis for ARHL and vestibular outcomes was not feasible due to substantial methodological heterogeneity (6, 8, 10, 19, 21- 23; see Table 1), therefore a narrative synthesis has been provided. Available studies examined the association between HL and vestibular subsites degeneration. HL on high frequencies seemed to be significantly related to saccular dysfunction (Agrawal et al., 2009). As previously reported, cochlea and saccule embryologically develop in the membranous labyrinth from the same origin and the inferior vestibular nerve innervates this anatomical area: a similar pattern of degeneration in cells of spiral and vestibular ganglions has also been found (Gluth and Nelson, 2017).

In clinical practice, lots of patients with different types of presbycusis suffer from vestibular dysfunction. However, it is unclear whether there is an actual causative association, or just a coexistence of age-related changes in the central nervous system, leading to an impairment of both systems. In the Kurtaran et al. (Kurtaran et al., 2016) population, HL was associated with reduction of amplitudes and prolonged P1 and N1 latency periods in VEMPs, thus suggesting a possible correlation between ARHL and peripheral vestibular deficit (Kurtaran et al., 2016). This supported the hypothesis that vestibular weakness can accompany HL, even without predisposing factors for vestibular diseases (Kurtaran et al., 2016). On the contrary, Onn et al. (Onn et al., 2021) and Teplitxky et al. (Teplitxky et al., 2023) did not find a significant association between vestibular dysfunction and presbycusis. Thus, we highlight the need for further research with standardized protocols to shed light on the interplay between ARHL and age-related vestibular loss (Paplou et al., 2023).

### 4.2 ARHL effects on gait and falls

ARHL has been increasingly recognized as a factor that might negatively influence gait and balance in older adults. Hearing contributes to spatial orientation and environmental awareness, both of which are essential for static and adaptive gait (Lin and Ferrucci, 2012).

In the meta-analysis of six studies, ARHL appeared associated with poorer TUG performance (see Figure 2) but meta-regression indicated that age imbalance fully explained this pattern (Figure 3), leaving no residual association. Moreover, according to Figure 4, a robust, clinically relevant association between ARHL and incident falls was found. It should be noted that our retrieved OR of 1.55 corresponds to RR ≈ 1.35 when the baseline 1-year fall risk is 27.6% (community-dwelling older adults) and RR ≈ 1.25 when the baseline risk is 43% (higher-risk cohorts), still consistent with a meaningful contribution of hearing status to fall propensity (Mahmoudzadeh Khalili et al., 2024; World Health Organization, 2007). Overall, the included studies have shown that subjects with ARHL had slower walking speeds, shorter stride lengths, and increased gait variability, in comparison to age-matched controls (Sakurai et al., 2021; Viljanen et al., 2009b; Li et al., 2013); these conditions were all associated with a higher risk of falls (Viljanen et al., 2009a; Chongvisal et al., 2019; Kamil et al., 2016; Mamo et al., 2023; Sakurai et al., 2021; Sakurai et al., 2022; Sakurai et al., 2025a; Lin and Ferrucci, 2012). In 2009, a prospective cohort-study firstly explored the relationship between hearing acuity and walking difficulties in older women, by measuring walking speed, walking endurance and self-report difficulties over a 2-km distance (Viljanen et al., 2009b). Poorer hearing levels were significantly associated with reduced balance and higher risk of falls, which could have a direct effect on mobility decline and hence on quality of life of aging people (Viljanen et al., 2009b). Other studies strengthen the association between HL and gait impairment by gait speed analysis (Kolasa et al., 2024; Sakurai et al., 2021; Sakurai et al., 2022; Sakurai et al., 2025a; Wollesen et al., 2018). Sakurai et al. (Sakurai et al., 2021) explored the relationship between ARHL and gait variability: poorer hearing acuity was significantly associated with slower gait speed and increased stride length variability, whereas stride time variability was not significantly affected. Among subjects with HL, those reporting multiple falls had higher stride length variability, but not necessarily slower gait or changes in stride time variability compared to non-fallers. These findings suggested that auditory deficits might compromise spatial consistency in gait (i.e., stride length), increasing the risk of falls, even in the absence of marked reductions in temporal gait variability (i.e., stride time) (Sakurai et al., 2021). Moreover, Sakurai (Sakurai et al., 2022) reported how ARHL and gait performance interacted to affect both global cognition and the risk of fall among the older persons. Slow gait in combination with moderate HL exhibited significantly lower cognitive assessment scores (Montreal Cognitive Assessment-MoCA test) and higher incidence of falls (Sakurai et al., 2022). Conversely, older adults with moderate HL but preserved gait speed did not show these deficits (Sakurai et al., 2022). In addition, the simultaneous presence of ARHL and slow gait markedly increased the risk of single and recurrent falls as well as fall-induced fractures and minor injuries, compared to when these risk factors were present separately (Sakurai et al., 2025a).

Additionally, the TUG test (Podsiadlo and Richardson, 1991) was used to evaluate dynamic balance abilities in older adults. Koh et al. (Koh et al., 2015) observed that the TUG time increased as age increased, even if the comparison of the results between the normal hearing and HL groups revealed there was no significant difference in the TUG times. However, in daily life, balance and walking function mostly occur in the form of multi-tasking phenomena, thus the TUG itself can be misleading in assessing effective functional mobility. Cosetti et al. (Cosetti et al., 2025) applied a dual-task paradigm to the instrumented TUG (iTUG) test, to investigate differences in dynamic balance between older adults with bilateral HL and age-matched normal hearing controls. The total duration of iTUG test did not differ significantly between the two groups, whereas the sub-component analysis of the test did. ARHL subjects showed prolonged sit-to-stand and stand-to-sit times in comparison to the normal hearing cohort (Cosetti et al., 2025). These findings suggested that, while overall mobility might appear comparable, there could be differences in anticipatory balance control in older adults with HL, who also showed specific impairment at the conclusion phase of the task, particularly under cognitive load (dual task). This might reflect a reduced attentional capacity, where added cognitive demand due to the HL disproportionately affected components of gait, potentially impacting on the risk of falling (Cosetti et al., 2025). Furthermore, in the dual-task TUG test combined with cognitive forward and backward digit span tasks, Söylemez et al. (Soylemez et al., 2024) found that HL severity was significantly associated with reduced working memory, and the performance was impaired in presbycusis cases compared to younger controls. Notably, when presbycusis subjects were divided into HA-users and non-users, the former performed significantly better on the cognitive components of the dual-task tests than the latter, suggesting that amplification may reduce listening-related cognitive load and help to preserve cognitive-motor integration during gait.

The Oklahoma Longitudinal Assessment of the Health Outcomes of Mature Adults (OKLAHOMA) prospective studies cohort (Mold et al., 2024) highlighted that participants with moderate or severe ARHL and those with peripheral neuropathy (PN) showed significantly poorer balance and increased gait time, indicating impaired locomotor performance among affected older patients. ARHL and PN independently predict earlier mortality, but their combination reduces survival time more than either sensory deficit alone. This additive effect of ARHL and PN can be mediated in part through impaired balance and gait and underscores the importance of evaluating both sensory systems in older persons people, in order to promptly identify the conditions and pursue prevention and treatment strategies that may help improve longevity in this population (Mold et al., 2024).

Kolasa et al. (2024) examined if HL was independently associated with physical performance, postural sway and gait variability. By using the Short Physical Performance Battery (SPPB) – that evaluates lower-body strength, balance and gait speed–the force platform and the accelerometer, Kolasa et al. (2024) provided a comprehensive and multidimensional assessment of physical function which is essential for everyday activities. They confirmed the previously reported (Kolasa et al., 2023) significant negative association between HL and SPPB total score, suggesting that people with HL had a worse physical performance and could face an increased risk of falls (Kolasa et al., 2023; Kolasa et al., 2024). A significant association between HL and increased postural sway on a firm surface with open and closed eyes, as well as on a foam surface with eyes closed, was found (Kolasa et al., 2024), in line with what has already been reported by other studies (Berge et al., 2019; Agmon et al., 2017). The Kolasa et al. (2024) study also showed that patients with HL had a greater gait variability during dual-task walking across all directions in comparison to individuals with normal hearing, supporting the Sakurai et al. (2021) findings.

Evaluating the effects of HL on dual-task performance while walking, Lau et al. (2016), found that older adults with bilateral HL showed more overall stride time variability than the normal-hearing group. Again, these findings suggested that ARHL might compromise standing balance and walking performance, particularly under cognitively demanding conditions like daily activities, and might serve as an early marker for mobility decline and fall risk in the aging population. The association between ARHL and risk of falls was also assessed and confirmed by other studies (Viljanen et al., 2009a; Chongvisal et al., 2019; Kamil et al., 2016; Mamo et al., 2023; Sakurai et al., 2021; Sakurai et al., 2022; Sakurai et al., 2025a) using appropriate clinical scales, such as the Activities-Specific Balance confidence scale (Morris et al., 2023; Bruce et al., 2019a; Cosetti et al., 2025), the Dynamic Gait Index (Bruce et al., 2019a), the Falls Efficacy Scale International (FES-I), and a self-reported fall frequency (Viljanen et al., 2009a; Chongvisal et al., 2019; Kamil et al., 2016; Mamo et al., 2023; Sakurai et al., 2021; Sakurai et al., 2022; Sakurai et al., 2025a). On the contrary, Purchase-Helzner (Purchase-Helzn et al., 2004) found that neither the age-adjusted annual fall rate nor the risk of incident fracture differed significantly by hearing category.

As already mentioned, besides the improvement of postural stability by a fixed sound source, Basta et al. (2023) observed that continuous auditory information at low or moderate intensities seemed to be significantly helpful in maintaining postural control during easy and challenging walking. This could further promote the importance of auditory rehabilitation in people with ARHL for improving static and dynamic gait control, as discussed in the subsequent section.

### 4.3 Effects of hearing rehabilitation on vestibular system and gait

Available results supported the hypothesis that HA could positively influence balance and postural control, as shown in Table 2. In line with the well-known effective role of auditory input on dynamic balance and gait, the studies showed that HA-users exhibited better dual-task walking performance and visual working memory, thus indicating the auditory rehabilitation’s protective effect against falls in cognitively demanding situations (Soylemez et al., 2024).

As a matter of fact, Picciotti et al. (2024) found that, after 12 months of HA fitting, patients with presbycusis and presby-vestibulopathy improved in their balance scales and dizziness-related handicap scores, reducing their fall risk. Similar results were later reproduced by Sakurai et al. (2025b). Moreover, a dose-response relationship between HA use and fall prevention was suggested by Campos et al. (2023), who detected significantly lower odds of falling in consistent HA-users. Overall, the current evidence suggests that HA provides measurable benefits in postural control by restoring auditory spatial cues, improving sensory integration, and facilitating vestibular compensation. Nonetheless, the heterogeneity across investigations (Table 3) reflects the different study design and patient-level moderators: balance gains from HA are related to HL severity, coexisting vestibular dysfunction, and device use/adherence. Nevertheless, the overall evidence supports incorporating auditory assessment and rehabilitation within multifactorial fall-prevention programmes for older adults. Accordingly, our meta-analysis indicated small-to-moderate, consistent improvements in posturography with HA ON vs. OFF, particularly when visual and somatosensory cues were impoverished, supporting a role for auditory input in sensory reweighting (See Figure 5).

Concerning cochlear implantation, we found no specific study addressing its impact on posture, gait and risk of falls. Nayak et al. (Nayak et al., 2022) performed CI ipsilateral to the reduced vestibular response in 67.4% of cases and contralateral in 32.6%, with no significant difference observed in postoperative dizziness rates between these groups, highlighting that vestibular dysfunction was more strongly associated with abnormal preoperative videonystagmography results or existing vestibular conditions. Although it is plausible that CI, as well as HA, may have a positive effect on posture, gait and risk of falls protection, this hypothesis needs to be confirmed in future research. In CI candidates, a thorough preoperative vestibular assessment and individualized risk evaluation remain essential in order to optimize hearing rehabilitation outcomes while safeguarding vestibular health.

Regarding non-auditory rehabilitation interventions, Bruce et al. (2019b) and Prasansuk et al. (2004) showed how training specificity shapes balanced outcomes in ARHL. In Bruce’s clinical trial (Bruce et al., 2019a), combined cognitive plus aerobic training sequentially improved chair-rise performance but did not enhance standing balance on posturography. By contrast, Prasansuk’s clinical trial applied a 20-week Cawthorne–Cooksey vestibular programme and demonstrated posturographic balance gains with high acceptability (Prasansuk et al., 2004). Consequently, when the clinical target is postural stability and fall risk, interventions should prioritize task-specific vestibular and sensory-integration training, with general aerobic/cognitive conditioning used as an adjunct rather than a substitute.

### 4.4 Strengths, limits, and future directions

The main limitation of this systematic review and meta-analysis may reside in the heterogeneity of study designs, measures and evaluation methods across the included articles.

Instead, the primary strength lies in the article selection process, with circumscribed inclusion/exclusion criteria, allowing for the inclusion of original articles with an overall low-to-moderate risk of bias (see also Tables 1-3). Moreover, beside a comprehensive qualitative evaluation of the state of knowledge on the relationship between ARHL and vestibular impairment, a quantitative meta-analysis regarding TUG performance between individuals with ARHL and controls was performed (also formally investigating the possible confounding effects) as well as a meta-analysis of fall risk in ARHL vs. age-matched NH. In addition, the within-participant analysis allowed us to quantitatively explore the effect of HA in terms of posturography outcomes. Further studies are mandatory to elucidate in detail the possible clinical implications of further acoustic rehabilitation strategies (e.g., incorporating auditory cues in rehabilitation or training programmes) on gait in older persons, to possibly enhance postural stability and reduce fall risk.

## 5 Conclusion

In this systematic review, a qualitative synthesis showed frequent associations between ARHL and vestibular dysfunction. In quantitative analysis, ARHL was linked to impaired balance and mobility, but that part of the observed deficit reflected residual confounding by age. A greater gait variability in dual-task conditions and heightened fall risk among the ARHL population was also reported. Moreover, HA intervention may lead to better static posturography measures. Taken together, these findings suggested that while chronological age remained a powerful driver of mobility outcomes, ARHL contributed additional, modifiable risk through sensory and cognitive-motor pathways, and that rehabilitation may offer clinically meaningful gains in postural control within multimodal fall-prevention strategies.

The importance of balance in impacting the quality of life of individuals with HL should not be underestimated, as balance is essential for most daily activities. By 2060, nearly a quarter of the U.S. population will be aged over 65 years, emphasizing the growing public health concerns related to falls and associated injuries (Riska et al., 2022). Balance impairments and resulting falls significantly impact morbidity and mortality, increasing hospitalization risk tenfold and accounting for over 50% of accidental deaths among the older persons (Paplou et al., 2023). The economic burden associated with falls due to sensory decline further underscores the importance of early detection and multidisciplinary interventions involving various specialists such as audiologists, ENT, neurologists, cardiologists, geriatricians, including combined audiological and vestibular rehabilitation programmes to enhance functional independence and reduce healthcare costs.

## References

[B1] Agmon M. Lavie L. Doumas M. (2017). The association between hearing loss, postural control, and mobility in older adults: a systematic review. J. Am. Acad. Audiology 28 (6), 575–588. 10.3766/jaaa.16044 28590900

[B2] Agrawal Y. Carey J. P. Della Santina C. C. Schubert M. C. Minor L. B. (2009). Disorders of balance and vestibular function in US adults: data from the national Health and Nutrition Examination Survey, 2001-2004. Arch. Intern Med. 169 (10), 938–944. 10.1001/archinternmed.2009.66 19468085

[B3] Azevedo C. Vilarinho S. Sousa Menezes A. Milhazes Mar F. Dias L. (2022). Vestibular and cochlear dysfunction in aging: two sides of the same coin? World J. Otorhinolaryngol. Head. Neck Surg. 8 (4), 308–314. 10.1002/wjo2.59 36474668 PMC9714048

[B4] Basta D. Borsellino L. Anton K. Ernst A. (2023). Influence of auditory information on postural control during different gait tasks in the elderly. J. Int. Adv. Otol. 19, 22–27. 10.5152/iao.2023.22671 36718032 PMC9984904

[B5] Behtani L. Paromov D. Moïn-Darbari K. Houde M. S. Bacon B. A. Maheu M. (2024). Hearing aid amplification improves postural control for older adults with hearing loss when other sensory cues are impoverished. Trends hear. 28, 23312165241232219. 10.1177/23312165241232219 38356376 PMC10868491

[B6] Berge J. E. Nordahl S. H. G. Aarstad H. J. Goplen F. K. (2019). Hearing as an independent predictor of postural balance in patients evaluated for dizziness. Otolaryngol. Head. Neck Surg. 161 (3), 478–484. 10.1177/0194599819844961 31013210

[B7] Brown C. Tollefson N. Dunn W. Cromwell R. Filion D. (2001). The adult sensory profile: measuring patterns of sensory processing. Am. J. Occup. Ther. 55 (1), 75–82. 10.5014/ajot.55.1.75 11216370

[B8] Bruce H. Lai L. Bherer L. Lussier M. St-Onge N. Li K. Z. H. (2019a). The effect of simultaneously and sequentially delivered cognitive and aerobic training on mobility among older adults with hearing loss. Gait Posture 67, 262–268. 10.1016/j.gaitpost.2018.10.020 30390596

[B9] Bruce H. Aponte D. St-Onge N. Phillips N. Gagné J. P. Li K. Z. H. (2019b). The effects of age and hearing loss on dual-task balance and listening. J. Gerontol. B Psychol. Sci. Soc. Sci. 74 (2), 275–283. 10.1093/geronb/gbx047 28486677 PMC6327658

[B10] Campos L. Prochazka A. Anderson M. Kaizer A. Foster C. Hullar T. (2023). Consistent hearing aid use is associated with lower fall prevalence and risk in older adults with hearing loss. J. Am. Geriatr. Soc. 71 (10), 3163–3171. 10.1111/jgs.18461 37314100 PMC10592632

[B11] Chongvisal S. Supavanich W. Thongyai K. Prakairungthong S. Atipas S. Limviriyakul S. (2019). Hearing and balance survey in Thai elders. Siriraj Med. J. 71 (2), 131–142. 10.33192/smj.2019.21

[B12] Ciquinato D. S. A. Doi M. Y. Silva R. A. D. Oliveira M. R. Gil A. W. O. Marchiori L. L. M. (2020). Posturographic analysis in the elderly with and without sensorineural hearing loss. Int. Arch. Otorhinolaryngol. 24 (4), e496–e502. 10.1055/s-0040-1701271 33133269 PMC7593116

[B13] Cosetti M. Arie L. Kelly J. Ren J. Lubetzky A. V. (2025). Dual task iTUG to investigate increased fall risk among older adults with bilateral hearing loss. Am. J. Otolaryngol. 46 (1), 104536. 10.1016/j.amjoto.2024.104536 39662103

[B14] de Lima J. P. Manrique-Huarte R. Ferran S. Mallmann F. Gil D. C. Barrenechea B. A. (2024). Hearing and balance in healthy aging project: characterization of hearing, balance, and other associated disorders in three population groups aged 55 and over. Audiol. Neurootol 29 (4), 306–321. 10.1159/000536531 38447542 PMC11309056

[B15] Ferrucci L. Schrack J. A. Knuth N. D. Simonsick E. M. (2012). Aging and the energetic cost of life. J. Am. Geriatr. Soc. 60 (9), 1768–1769. 10.1111/j.1532-5415.2012.04102.x 22985146 PMC3569852

[B16] Foster J. I. Williams K. L. Timmer B. H. B. Brauer S. G. (2022). The association between hearing impairment and postural stability in older adults: a systematic review and meta-analysis. Trends 26, 23312165221144155. 10.1177/23312165221144155 36524292 PMC9761226

[B17] Gabriel G. A. Harris L. R. Gnanasegaram J. J. Cushing S. L. Gordon K. A. Haycock B. C. (2022). Vestibular perceptual thresholds in older adults with and without age-related hearing loss. Ear Hear 43 (2), 420–435. 10.1097/AUD.0000000000001118 34534156

[B18] Gluth M. B. Nelson E. G. (2017). Age-related change in vestibular ganglion cell populations in individuals with presbycusis and normal hearing. Otol. Neurotol. 38 (4), 540–546. 10.1097/MAO.0000000000001325 28125514

[B19] Gorecka M. M. Vasylenko O. Waterloo K. Rodríguez-Aranda C. (2021). Assessing a sensory-motor-cognition triad in amnestic mild cognitive impairment with dichotic listening while walking: a dual-task paradigm. Front. Aging Neurosci. 13, 718900. 10.3389/fnagi.2021.718900 34867267 PMC8633416

[B20] Ivanenko Y. Gurfinkel V. S. (2018). Human postural control. Front. Neurosci. 12, 171. 10.3389/fnins.2018.00171 29615859 PMC5869197

[B21] Jiam N. T. Li C. Agrawal Y. (2016). Hearing loss and falls: a systematic review and meta-analysis. Laryngoscope 126 (11), 2587–2596. 10.1002/lary.25927 27010669

[B22] Kamil R. J. Betz J. Powers B. B. Pratt S. Kritchevsky S. Ayonayon H. N. (2016). Health ABC study. Association of hearing impairment with incident frailty and falls in older adults. J. Aging Health 28 (4), 644–660. 10.1177/0898264315608730 26438083 PMC5644033

[B23] Kim G. Y. Cho Y. S. Jo M. Yun H. J. Quilter M. Kim D. Y. (2025). Effects of hearing aids on static and subjective balance in patients with hearing loss: a pilot study. J. Audiol. Otol. 29 (1), 8–12. 10.7874/jao.2024.00150 39916395 PMC11824520

[B24] Koh D. H. Lee J. D. Lee H. J. (2015). Relationships among hearing loss, cognition and balance ability in community-dwelling older adults. J. Phys. Ther. Sci. 27 (5), 1539–1542. 10.1589/jpts.27.1539 26157259 PMC4483437

[B25] Kolasa S. Bogen B. Nilsen R. M. Nordahl S. H. G. Goplen F. K. Engdahl B. (2023). Hearing threshold and physical performance in older people: a cross-sectional study from the HUNT4 cohort. Eur. Geriatr. Med. 14 (1), 165–172. 10.1007/s41999-022-00713-6 36396826 PMC9902320

[B26] Kolasa S. Magnussen L. H. Nilsen R. M. Wilhelmsen K. T. Goplen F. K. Nordahl S. H. G. (2024). Walking and balance in older adults with age-related hearing loss: a cross-sectional study of cases and matched controls. Gait Posture 113, 398–406. 10.1016/j.gaitpost.2024.07.301 39088930

[B27] Kurtaran H. Acar B. Ocak E. Mirici E. (2016). The relationship between senile hearing loss and vestibular activity. Braz J. Otorhinolaryngol. 82 (6), 650–653. 10.1016/j.bjorl.2015.11.016 26997575 PMC9444785

[B28] Kutlu S. Aydogan Z. Baş B. Meço C. Tokgoz-Yilmaz S. (2024). Interaction of sensory processing and balance in adult cochlear implant users. Eur. Arch. Otorhinolaryngol. 281 (11), 5651–5656. 10.1007/s00405-024-08776-w 38977472 PMC11512880

[B29] Lau S. T. Pichora-Fuller M. K. Li K. Z. Singh G. Campos J. L. (2016). Effects of hearing loss on dual-task performance in an audiovisual virtual reality simulation of listening while walking. J. Am. Acad. Audiol. 27 (7), 567–587. 10.3766/jaaa.15115 27406663

[B30] Lavie L. Tobia N. Slav-Zarfati N. Castel S. Banai K. (2024). Are current data sufficient to infer that hearing aids contribute to postural control and balance in older adults? A systematic review. Folia Phoniatr. Logop. 76 (3), 232–244. 10.1159/000534164 37717567 PMC11151983

[B31] Li L. Simonsick E. M. Ferrucci L. Lin F. R. (2013). Hearing loss and gait speed among older adults in the United States. Gait Posture 38 (1), 25–29. 10.1016/j.gaitpost.2012.10.006 23177614 PMC3845825

[B32] Lin F. R. Ferrucci L. (2012). Hearing loss and falls among older adults in the United States. Arch. Intern Med. 172 (4), 369–371. 10.1001/archinternmed.2011.728 22371929 PMC3518403

[B33] Mahmoudi E. Basu T. Langa K. McKee M. M. Zazove P. Alexander N. (2019). Can hearing aids delay time to diagnosis of dementia, depression, or falls in older adults? J. Am. Geriatr. Soc. 67 (11), 2362–2369. 10.1111/jgs.16109 31486068

[B34] Mahmoudzadeh Khalili S. Simpkins C. Yang F. (2024). A meta-analysis of fall risk in older adults with Alzheimer's disease. J. Am. Med. Dir. Assoc. 25 (5), 781–788.e3. 10.1016/j.jamda.2024.01.005 38378160 PMC11065606

[B35] Mamo S. K. Pearlman J. Wheeler K. A. (2023). Associations between age-related hearing loss, cognitive impairment, and multiple chronic conditions in a group care setting. J. Speech Lang. Hear Res. 66 (12), 5087–5108. 10.1044/2023_JSLHR-23-00067 37934882 PMC11001376

[B36] Marioni G. (2022). Vestibular rehabilitation for unilateral peripheral vestibular deficits. JAMA Otolaryngol. Head. Neck Surg. 148 (5), 434–435. 10.1001/jamaoto.2022.0158 35357407

[B37] Marioni G. Fermo S. Lionello M. Fasanaro E. Giacomelli L. Zanon S. (2013). Vestibular rehabilitation in elderly patients with central vestibular dysfunction: a prospective, randomized pilot study. Age (Dordr) 35 (6), 2315–2327. 10.1007/s11357-012-9494-7 23179254 PMC3825000

[B38] Mohanathas N. Montanari L. Gabriel G. A. Downey R. Li K. Z. H. Campos J. L. (2024). Realistic dual-task listening-while-balancing in older adults with normal hearing and hearing loss with and without hearing aids. Sci. Rep. 14 (1), 28758. 10.1038/s41598-024-79933-8 39567644 PMC11579314

[B39] Mold J. W. Lawler F. H. Liao X. Bard D. E. (2024). Associations between hearing loss, peripheral neuropathy, balance, and survival in older primary care patients. J. Am. Geriatr. Soc. 72 (11), 3427–3436. 10.1111/jgs.19142 39143038

[B40] Morris B. Cosetti M. Kelly J. Yang J. Harel D. Medlin A. (2023). Differing postural control patterns in individuals with bilateral and unilateral hearing loss (2023). Am. J. Otolaryngol. 44 (4), 103866. 10.1016/j.amjoto.2023.103866 36989756 PMC10330028

[B41] Nayak N. Kellermeyer B. Dornton L. Heyd C. Kim C. S. Wazen J. J. (2022). Vestibular dysfunction in cochlear implant candidates: prevalence and outcomes. Am. J. Otolaryngol. 43 (1), 103171. 10.1016/j.amjoto.2021.103171 34509078

[B42] Negahban H. Bavarsad Cheshmeh Ali M. Nassadj G. (2017). Effect of hearing aids on static balance function in elderly with hearing loss. Gait Posture 58, 126–129. 10.1016/j.gaitpost.2017.07.112 28772132

[B43] Onn M. A. Daud K. M. Salim R. (2021). Prevalence of vestibular dysfunction with presbycusis among elderly in Malaysia. Pak J. Med. Health Sci. 15, 3478–3480. 10.53350/pjmhs2115123478

[B44] Paplou V. G. Schubert N. M. A. van Tuinen M. Vijayakumar S. Pyott S. J. (2023). Functional, morphological and molecular changes reveal the mechanisms associated with age-related vestibular loss. Biomolecules 13 (9), 1429. 10.3390/biom13091429 37759828 PMC10526133

[B45] Picciotti P. M. Di Cesare T. Asprella F. A. Asprella G. A. Paludetti G. Galli J. (2024). Presbycusis and presbyvestibulopathy: balance improvement after hearing loss restoration. Hear Balance Commun. 22 (3), 94–99. 10.4103/HBC.HBC_25_24

[B46] Podsiadlo D. Richardson S. (1991). The timed “Up and Go”: a test of basic functional mobility for frail elderly persons. J. Am. Geriatr. Soc. 39 (2), 142–148. 10.1111/j.1532-5415.1991.tb01616.x 1991946

[B47] Prasansuk S. Siriyananda C. Nakorn A. N. Atipas S. Chongvisal S. (2004). Balance disorders in the elderly and the benefit of balance exercise. J. Med. Assoc. Thai 87 (10), 1225–1233. 15560702

[B48] Purchase-Helzner E. L. Cauley J. A. Faulkner K. A. Pratt S. Zmuda J. M. Talbott E. O. (2004). Hearing sensitivity and the risk of incident falls and fracture in older women: the study of osteoporotic fractures. Ann. Epidemiol. 14 (5), 311–318. 10.1016/j.annepidem.2003.09.008 15177269

[B49] Rafoul B. Tzemah-Shahar R. Lubetzky A. V. Cohen-Vaizer M. Karawani H. Agmon M. (2025). Effects of cochlear implantation on gait performance in adults with hearing impairment: a systematic review. PLoS One 20 (2), e0319322. 10.1371/journal.pone.0319322 40019924 PMC11870346

[B50] Riska K. M. Peskoe S. B. Kuchibhatla M. Gordee A. Pavon J. M. Kim S. E. (2022). Impact of hearing aid use on falls and falls-related injury: results from the health and retirement study. Ear Hear 43 (2), 487–494. 10.1097/AUD.0000000000001111 34334680 PMC9554784

[B51] Roth T. N. Hanebuth D. Probst R. (2011). Prevalence of age-related hearing loss in Europe: a review. Eur. Arch. Otorhinolaryngol. 268, 1101–1107. 10.1007/s00405-011-1597-8 21499871 PMC3132411

[B52] Rumalla K. Karim A. M. Hullar T. E. (2015). The effect of hearing aids on postural stability. Laryngoscope 125 (3), 720–723. 10.1002/lary.24974 25346316

[B53] Sakurai R. Suzuki H. Ogawa S. Takahashi M. Fujiwara Y. (2021). Hearing loss and increased gait variability among older adults. Gait Posture 87, 54–58. 10.1016/j.gaitpost.2021.04.007 33892392

[B54] Sakurai R. Kawai H. Yanai S. Suzuki H. Ogawa S. Hirano H. (2022). Gait and age-related hearing loss interactions on global cognition and falls. Laryngoscope 132 (4), 857–863. 10.1002/lary.29898 34636436

[B55] Sakurai R. Kawai H. Suzuki H. Ogawa S. Hirano H. Ito M. (2025a). Increased risk of falls in older adults with hearing loss and slow gait: results from the Otassha Study. Geroscience 47 (2), 2235–2244. 10.1007/s11357-024-01412-9 39485656 PMC11979016

[B56] Sakurai R. Nishinakagawa M. Hinakura K. Takahashi M. (2025b). Effects of wearing hearing aids on gait and cognition: a pilot study. Audiol. Neurootol 30 (4), 374–380. 10.1159/000544829 40024242

[B57] Sandoval-Lentisco A. López-López J. A. Sánchez-Meca J. (2024). Frequency of use of the revised Cochrane risk of Bias tool (RoB 2) in Cochrane and non-Cochrane systematic reviews published in 2023 and 2024. Res. Synth. Methods 15 (6), 1244–1245. 10.1002/jrsm.1755 39257179

[B58] Semenov Y. R. Bigelow R. T. Xue Q. L. du Lac S. Agrawal Y. (2016). Association between vestibular and cognitive function in U.S. adults. J. Gerontol. A Biol. Sci. Med. Sci. 71 (2), 243–250. 10.1093/gerona/glv069 26219850 PMC5864155

[B59] Soylemez E. Soylemez T. G. Apaydin A. S. Apaydin Z. K. Yasar M. (2024). The role of hearing aids in improving dual-task gait performance in older adults with presbycusis: a cognitive and motor analysis. Brain Behav. 14 (11), e70114. 10.1002/brb3.70114 39482836 PMC11527819

[B60] Szeto B. Zanotto D. Lopez E. M. Stafford J. A. Nemer J. S. Chambers A. R. (2021). Hearing loss is associated with increased variability in double support period in the elderly. Sensors (Basel) 21 (1), 278. 10.3390/s21010278 33406602 PMC7795333

[B61] Teplitxky A. Gautier J. Lievre M. Duval G. Annweiler C. Boucher S. (2023). Association between age-related hearing loss and gait disorders in older fallers. Aging Clin. Exp. Res. 35 (4), 785–791. 10.1007/s40520-023-02350-w 36786968

[B62] Vallamchetla S. K. Abdelkader O. Elnaggar A. Ramadan D. Islam Shourav M. M. Riaz I. B. (2025). Do it faster with PICOS: generative AI-Assisted systematic review screening. J. Biomed. Inf. 168, 104860. 10.1016/j.jbi.2025.104860 40447171

[B63] Viljanen A. Kaprio J. Pyykkö I. Sorri M. Koskenvuo M. Rantanen T. (2009a). Hearing acuity as a predictor of walking difficulties in older women. J. Am. Geriatr. Soc. 57 (12), 2282–2286. 10.1111/j.1532-5415.2009.02553.x 19874410

[B64] Viljanen A. Kaprio J. Pyykkö I. Sorri M. Pajala S. Kauppinen M. (2009b). Hearing as a predictor of falls and postural balance in older female twins. J. Gerontol. A Biol. Sci. Med. Sci. 64 (2), 312–317. 10.1093/gerona/gln015 19182227 PMC2655032

[B65] Vitkovic J. Le C. Lee S. L. Clark R. A. (2016). The contribution of hearing and hearing loss to balance control. Audiol. Neurootol 21 (4), 195–202. 10.1159/000445100 27251708

[B66] Wang Y. Zhong M. Li Y. Liu Y. Tong B. Qiu J. (2024). Association between hearing loss, asymmetric hearing, and postural instability. Ear Hear 45 (4), 827–836. 10.1097/AUD.0000000000001474 38351499

[B67] Wollesen B. Scrivener K. Soles K. Billy Y. Leung A. Martin F. (2018). Dual-task walking performance in older persons with hearing impairment: implications for interventions from a preliminary observational study. Ear Hear 39 (2), 337–343. 10.1097/AUD.0000000000000489 28857786

[B68] World Health Organization (2007). WHO global report on Falls prevention in older Age. Geneva, Switzerland: World Health Organization.

[B69] Wunderlich A. Wollesen B. Asamoah J. Delbaere K. Li K. (2024). The impact of cognitive-motor interference on balance and gait in hearing-impaired older adults: a systematic review. Eur. Rev. Aging Phys. Act. 21 (1), 17. 10.1186/s11556-024-00350-x 38914940 PMC11194914

[B70] Zuniga M. G. Dinkes R. E. Davalos-Bichara M. Carey J. P. Schubert M. C. King W. M. (2012). Association between hearing loss and saccular dysfunction in older individuals. Otol. Neurotol. 33 (9), 1586–1592. 10.1097/MAO.0b013e31826bedbc 23064383 PMC3498596

